# Multiple Sclerosis: MicroRNA Expression Profiles Accurately Differentiate Patients with Relapsing-Remitting Disease from Healthy Controls

**DOI:** 10.1371/journal.pone.0007440

**Published:** 2009-10-13

**Authors:** Andreas Keller, Petra Leidinger, Julia Lange, Anne Borries, Hannah Schroers, Matthias Scheffler, Hans-Peter Lenhof, Klemens Ruprecht, Eckart Meese

**Affiliations:** 1 febit biomed gmbh, Heidelberg, Germany; 2 Department of Human Genetics, Medical School, Saarland University, Homburg, Germany; 3 Center for Bioinformatics, Saarland University, Saarbruecken, Germany; 4 Biomarker Discovery Center Heidelberg, Heidelberg, Germany; 5 Department of Neurology, Charité Universitätsmedizin Berlin, Berlin, Germany; Institute of Infectious Disease and Molecular Medicine, South Africa

## Abstract

Multiple sclerosis (MS) is a chronic inflammatory demyelinating disease of the central nervous system, which is heterogenous with respect to clinical manifestations and response to therapy. Identification of biomarkers appears desirable for an improved diagnosis of MS as well as for monitoring of disease activity and treatment response. MicroRNAs (miRNAs) are short non-coding RNAs, which have been shown to have the potential to serve as biomarkers for different human diseases, most notably cancer. Here, we analyzed the expression profiles of 866 human miRNAs. In detail, we investigated the miRNA expression in blood cells of 20 patients with relapsing-remitting MS (RRMS) and 19 healthy controls using a human miRNA microarray and the Geniom Real Time Analyzer (GRTA) platform. We identified 165 miRNAs that were significantly up- or downregulated in patients with RRMS as compared to healthy controls. The best single miRNA marker, hsa-miR-145, allowed discriminating MS from controls with a specificity of 89.5%, a sensitivity of 90.0%, and an accuracy of 89.7%. A set of 48 miRNAs that was evaluated by radial basis function kernel support vector machines and 10-fold cross validation yielded a specificity of 95%, a sensitivity of 97.6%, and an accuracy of 96.3%. While 43 of the 165 miRNAs deregulated in patients with MS have previously been related to other human diseases, the remaining 122 miRNAs are so far exclusively associated with MS. The implications of our study are twofold. The miRNA expression profiles in blood cells may serve as a biomarker for MS, and deregulation of miRNA expression may play a role in the pathogenesis of MS.

## Introduction

Multiple sclerosis (MS) is a chronic inflammatory demyelinating disease of the brain and spinal cord, primarily affecting young adults [1]. It is widely believed that MS is an immune-mediated disease whose clinical manifestations and course, as well as response to therapy appear to be heterogeneous, as may be the underlying pathogenic mechanisms [1]. Biomarkers are defined as parameters that can be objectively measured and evaluated as indicators of pathogenic processes or responses to a therapeutic intervention [2]. Identification of reliable biomarkers for MS bears the potential for an improved diagnosis of MS, monitoring of disease activity and progression, and evaluation of treatment responses. In recent years, the field of biomarker discovery has gradually shifted from the aim of finding a single perfect surrogate marker to the construction of composite markers with higher performance, taking advantage of technologies allowing unbiased screening, including microarray analyses. However, identification of suitable biomarker sets for MS based on parameters in peripheral blood is only in its infancy [2], [3].

MicroRNAs (miRNAs) are short (about 22 nucleotides in length) single-stranded regulatory RNAs that modulate gene expression at the posttranscriptional level by repressing translation of specific messenger RNA (mRNA) targets, eventually resulting in downregulation of protein expression. They play important roles in a variety of physiologic and pathologic processes, most notably oncogenesis [4], [5]. Furthermore, miRNAs are involved in the regulation of the immune system [6]. Evidence suggests that miRNAs are present in a remarkably stable form in human blood, where they occur as free circulating nucleic acids, in microvesicles, and in mononuclear blood cells [7], [8]. Recent proof-of-principle studies demonstrated that the analysis of miRNA expression in sera or blood cells may be a promising approach for blood-based diagnosis of a number of human cancers and also autoimmune diseases [8]–[10]. Those studies have also shown that global patterns of miRNA expression might be more revealing than analysis of single miRNAs. Together, the previous findings suggest that miRNA expression signatures in blood have the potential to serve as biomarkers for various human diseases.

Here, we investigated miRNA expression patterns in blood cells of patients with relapsing-remitting MS (RRMS), as compared to healthy controls, using a miRNA microarray which contains all 866 human miRNAs and miRNA star sequences deposited in the Sanger miRBase version 12.0 [http://microrna.sanger.ac.uk/sequences/, 11]. We thereby identified 165 significantly deregulated miRNAs in MS and demonstrate that unique miRNA signatures in patients with RRMS allow accurate differentiation from healthy controls. These data suggest that miRNA expression signatures may represent a potentially useful biomarker for the diagnosis of MS and that dysregulation of miRNA expression could play a role in the complex pathogenesis of MS.

## Results

### Altered miRNA expression in patients with relapsing-remitting MS

We analyzed the expression of 866 miRNAs and miRNA star sequences in blood cells of 20 patients with RRMS as well as 19 healthy controls. Demographic and clinical details of the study subjects are listed in Supplemental Table S1. Following RNA isolation and on-chip labeling of miRNAs, the miRNA expression profiles were measured using the Geniom Biochip miRNA homo sapiens and the Geniom Real Time Analyzer GRTA (febit GmbH, Heidelberg). Raw images were analyzed using the Geniom Wizard software and the intensity values were background subtracted and quantile normalized. To test the reproducibility of the platform we screened 8 technical replicates of purchased total RNA (Ambion). We determined a mean correlation value of 0.97 for the technical replicates. Measurement of biological replicates showed a mean correlation of 0.87 and a variance of 0.009.

**Figure 1:**
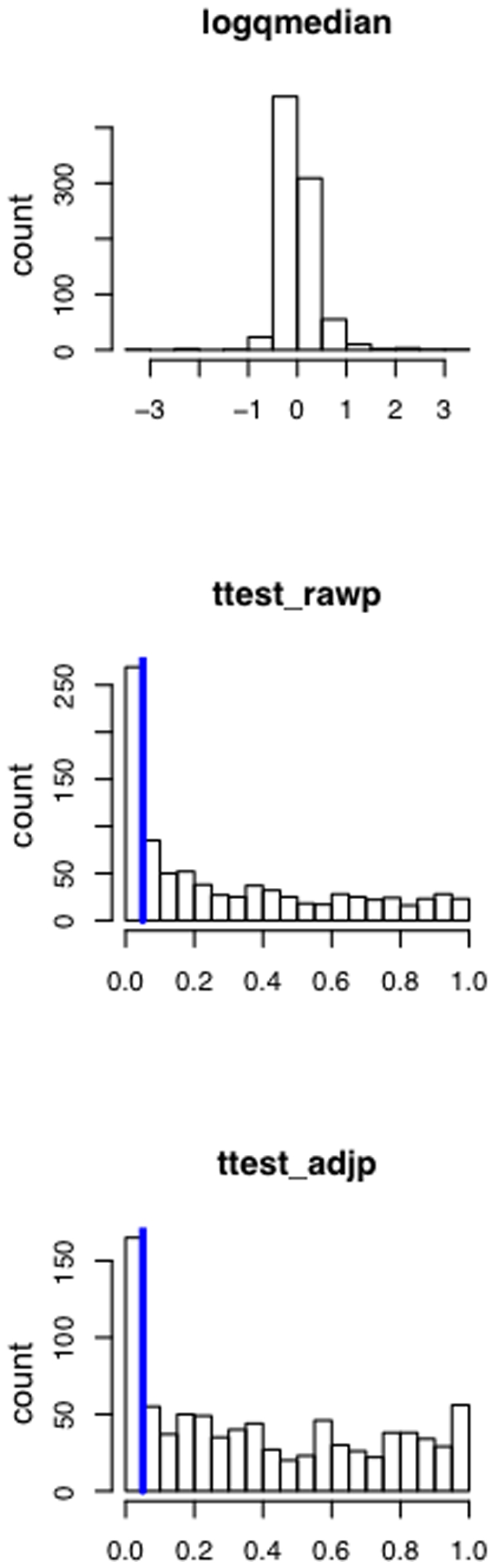
Histogram plot of the logarithm of fold changes, raw t-test p-values and adjusted p-values for all screened miRNAs. The upper part of the figure shows the logarithms of fold changes. These are almost normally distributed between -3 and 3. The middle and bottom part of the figure present histograms of adjusted p-values for the limma test and t-test. The t-test showed a slightly decreased number of significant miRNAs. Nevertheless, for both tests a clear tendency towards low p-values and thus high significance can be seen. The vertical blue line denotes the significance threshold of 0.05.

**Figure 2:**
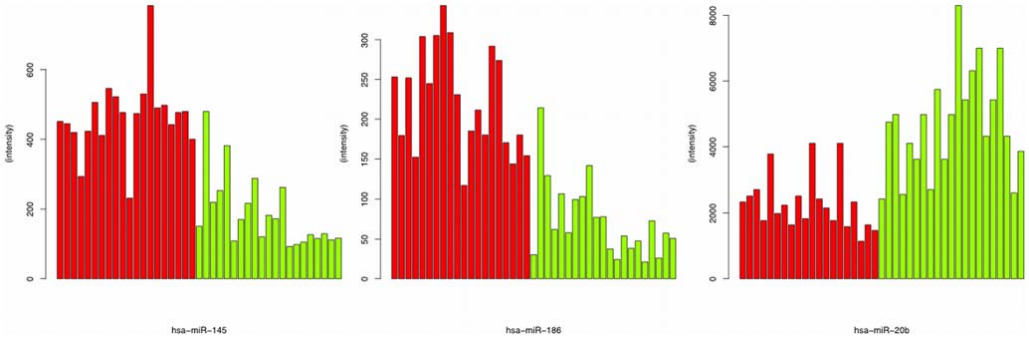
Barplots detailing the intensity values for the miRNAs hsa-miR-145 (a), hsa-mir-186 (b), and hsa-miR-20b (c). MS sera are indicated by red bars, control sera are indicated by green bars.

We next applied hypothesis testing to identify miRNAs deregulated in blood cells of MS patients as compared to controls. Following verification of an approximately normal distribution using Shapiro-Wilk test, we performed two-tailed unpaired t-tests for each miRNA. The respective p-values were adjusted for multiple testing by the Benjamini-Hochberg approach. In total, we detected 165 miRNAs significantly deregulated in blood cells of MS patients as compared to controls. Histogram plots of the logarithm of fold quotients, the raw t-test p-values and the adjusted p-values are presented in Figure 1. A complete list of deregulated miRNAs is given in Supplemental Table S1. From the 165 significantly deregulated miRNAs 74 (44.8%) were up-regulated and 91 (55.2%) were down-regulated. The ten miRNAs that were most significantly deregulated included hsa-miR-145 (1.46·10^−7^), hsa-miR-186 (2.89·10^−7^), hsa-miR-664 (5.25·10^−5^), hsa-miR-20b (1.48·10^−4^), hsa-miR-422a (1.48·10^−4^), hsa-miR-142-3p (1.54·10^−4^), hsa-miR-584 (1.56·10^−4^), hsa-miR-223 (1.63·10^−4^), hsa-miR-1275 (1.16·10^−4^) and hsa-miR-491-5p (2.83·10^−4^). Remarkably, 9 of the best ten miRNA biomarkers (90%) were significantly up-regulated in MS, while only one miRNA (hsa-miR-20b) was down-regulated. For the two best miRNAs, hsa-miR-186 and hsa-miR-145, we present bar plots showing the intensity values for all MS and control samples in Figures 2a and 2b. For the single down-regulated miRNA hsa-miR-20b, the bar plot is presented in Figure 2c. Table 1 shows the ten most deregulated miRNAs. To further validate our data, we performed RT-qPCR on two miRNAs that are up-regulated in our array profiles and are associated from literature to be disease related [12]. In detail, we exemplarily screened 4 diseased and 4 control samples of miRNAs has-let-7c and has-miR-233. miR-let-7c was up-regulated 1.57 fold in the arrays of the respective patients and the RT-qPCR showed a fold change of 1.76. For miR-223, the arrays showed a fold-change of 2.21 and the RT-qPCR of 1.84. We additionally computed receiver operator characteristic curves (ROC) for each of the best miRNAs together with the area under the curve value (AUC). The more the AUC differs from 0.5, the better a biomarker is. The most extreme values of the AUC are 0 and 1. A value of 1 means, that none of the control values exceeds any of the MS values. A value of 0 means, that none of the MS values exceeds any of the control values.

**Table 1:** The ten most significantly deregulated miRNAs.

	median MS	median normal	fold quotient	Logarithm of fold quotient	t-test adjusted p-values
hsa-miR-145	474,928	150,87	3,148	1,147	1,46E-07
hsa-miR-186	221,159	57,826	3,825	1,341	2,89E-07
hsa-miR-664	563,351	239,837	2,349	0,854	5,25E-05
hsa-miR-20b	2197,846	4746,011	0,463	−0,77	0,000148065
hsa-miR-422a	309,874	148,899	2,081	0,733	0,000148065
hsa-miR-142-3p	171,558	21,71	7,902	2,067	0,000154481
hsa-miR-584	266,721	76,725	3,476	1,246	0,000156481
hsa-miR-223	4131,063	1986,446	2,08	0,732	0,00016217
hsa-miR-1275	164,254	92,348	1,779	0,576	0,000163285
hsa-miR-491-5p	192,594	118,522	1,625	0,485	0,000283444

For the best miRNA, hsa-miR-145, we obtained an AUC value of 0.96. Using this miRNA we classified 35 (89.7%) of the 39 samples correctly, but four samples (9.8%) were misclassified (two MS sera false negatively, and two control sera false positively) leading to a specificity of 89.5%, a sensitivity of 90% and an accuracy of 89.7%. However, these results are not validated by a re-sampling technique as bootstrapping or cross-validation and are based only on a single marker.

### Evaluating complex fingerprints

In order to improve the statistical reliability we combined the predictive power of multiple miRNAs by using statistical learning techniques. In detail, we applied Support Vector Machines (SVM) with different kernels (linear, polynomial, sigmoid, radial basis function) to the data and carried out a hypothesis test based on subset selection as described in Material and Methods. To gain statistical significance we carried out 100 repetitions of standard 10-fold cross validation. Likewise, we computed 100 repetitions for the permutation tests where samples with randomly assigned class labels were classified.

**Figure 3:**
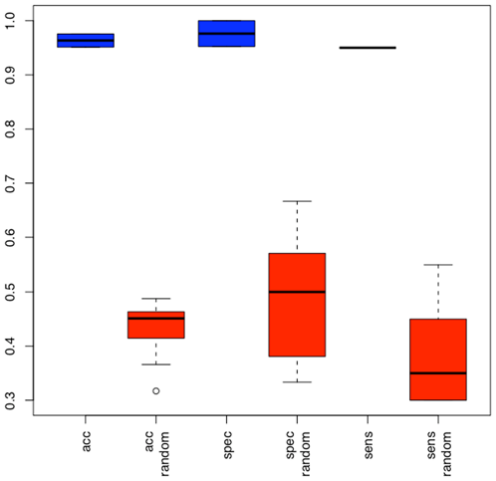
Boxplot of the classification results. The blue boxes show the classification accuracy, specificity and sensitivity over the repeated cross-validation for a subset of 48 miRNAs. The red boxes show the respective accuracy, specificity and sensitivity for permutation test.

**Figure 4:**
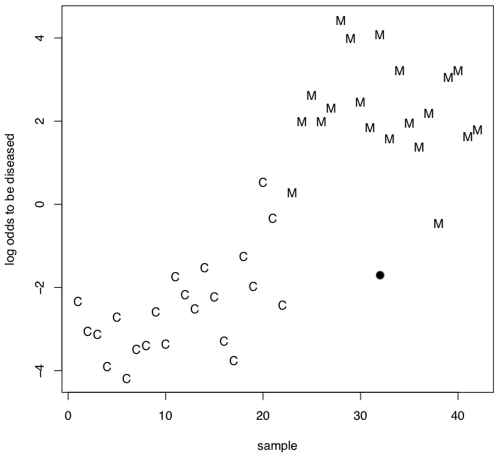
Exemplarily classification result. The logarithm of the quotient of the probability to be a MS sample and the probability to be a control sample for each control (C) and each MS (M) sample is given on the y-axis. If this quotient is greater than one (thus the logarithm greater zero) the sample is more likely to be a MS sample than a control sample.

The best results were obtained with radial basis function SVM and a subset of 48 miRNAs (see Supplemental Table S1). These miRNAs allowed the discrimination between blood samples of MS patients and blood samples of controls with an accuracy of 96.3%, a specificity of 95%, and a sensitivity of 97.6%. The permutation tests showed significantly decreased accuracy, specificity, and sensitivity rates, corresponding to random guessing. The classification results and the results of the permutation tests are shown in Figure 3. These results show that the obtained results are not due to an overfit of the statistical model on the miRNA fingerprints. Additionally, we present one randomly selected example of a classification in MS and controls in Figure 4. This graphic shows for each control (C) and each MS (M) sample the logarithm of the quotient of the probability to be a MS sample and the probability to be a control sample. The probabilities have been computed by the R implementation of the libsvm relying on the distance of the samples from the separating hyperplane. If the quotient of the probabilities is greater than one (thus the logarithm is greater zero) the sample is more likely to be a MS sample than a control sample. Figure 4 clearly outlines that in general MS samples have logarithmized quotients of greater 0 while control samples have quotients of below 0.

### No influence of age, gender, and MS therapy on miRNA expression profiles

To cross-check that age and gender did not influence our analysis, we computed t-tests using the normal samples. In case of males versus females we did not find any statistically significant deregulated miRNA. The most significant miRNA, hsa-miR-423, showed an adjusted significance level of 0.78. To test for the influence of donor age we compared the profiles obtained from old versus young patients by splitting the total group in half based on the age. Here, the most significant miRNA, hsa-miR-890, obtained an adjusted p-value of 0.87. As for gender, we did not find any deregulated miRNAs, thus providing evidence that age and gender do not have a substantial influence on the miRNA profiles. Additionally, we determined the influence of MS therapy by comparing the group of patients treated with glatiramer acetate (n = 9) to the group treated with interferon-β (n = 10). As for gender and age we did not find any significantly deregulated miRNAs between those two groups.

### MiRNA deregulated in patients with MS only partially overlap with miRNAs previously associated with other human diseases

**Figure 5:**
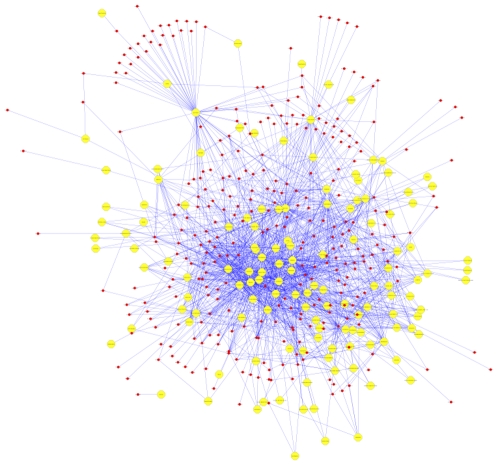
Complete disease network without MS. The network indicates human diseases by yellow nodes and miRNAs by red nodes. An edge between a disease and a miRNA indicates a miRNA that has previously been associated with the respective disease. This figure is available as DIN A0 Poster within the Supplemental Material (Supplemental Figure S1).

**Figure 6:**
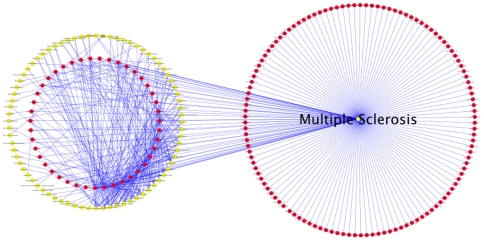
Multiple Sclerosis Network. This network indicates diseases by yellow nodes and miRNAs by red nodes. The network is restricted to the miRNAs significant for MS. Most of the miRNAs are associated with MS but not with other diseases, indicated by the red circles in the right part of this figure. This figure is available as DIN A0 Poster within the Supplemental Material (Supplemental Figure S2).

To examine if the detected 165 significantly deregulated miRNAs are already related to other human diseases we used the “Human microRNA Disease Database (HMDD)” that contains information on deregulated miRNA for over 100 human diseases. Altogether, over 2000 relations are included in the HMDD [12]. We created a bipartite graph were red nodes indicate miRNAs and yellow nodes indicate human diseases (see Figures 5 and 6). Edges between a miRNA and a disease indicate deregulated miRNAs in the respective disease.

We created a network containing 452 nodes, 137 of which belong to diseases and 315 to miRNAs. The network, which is shown in Figure 3 (an enlarged high-resolution poster version is available as Supplemental material to this manuscript), contained 1617 unique edges. Since MS is not included as disease in this network, we modified the network as follows: We added a disease node “Multiple Sclerosis” and created edges between this node and all significant deregulated miRNAs. Additionally, we removed all disease nodes that are not linked to any MS miRNA and all miRNAs belonging only to removed disease nodes. Our novel network, which is shown in Figure 4 thus contains only those miRNAs that are significant in MS and other diseases (middle part of Figure 4) and those that are significant in MS, only. This shrunken network contained 76 disease nodes together with the 165 significant miRNAs. Remarkably, only 43 of the 165 (26%) miRNAs were related to a disease other than MS while the remaining 122 (74%) miRNAs were only connected to MS. Of these 122 miRNAs, 33 (27%) were so-called mature star sequences. These results provide strong evidence that the detected complex miRNA profile appears to be rather specific for MS.

## Discussion

In this study, we identified 165 significantly deregulated miRNAs in blood cells of patients with RRMS compared to healthy controls. The most significant deregulated miRNA, hsa-miR-145, differentiated between MS patients and healthy controls with an accuracy, specificity, and sensitivity of 89.7%, 89.5%, and 90.0%, respectively. Using a set of 48 of the 165 significantly deregulated miRNAs, we were able to improve the classification results, reaching an accuracy of 96.3%, a specificity of 95%, and a sensitivity of 97.6%. Together, these data suggest that single miRNAs, and even more so miRNA expression profiles, may have the potential to serve as diagnostic biomarkers for RRMS.

Since our study includes patients with MS and healthy controls only, future investigations should corroborate the specificity of the observed miRNA expression patterns for MS by including groups of patients with other inflammatory neurological diseases. Comparison of the pattern of deregulated MS miRNAs with miRNAs previously found to be deregulated in other human diseases showed a remarkably high percentage of miRNAs (74%) that are deregulated in MS but not in any other disease. Notably, these diseases comprise neoplastic and degenerative neurological diseases as well as systemic inflammatory diseases (see Figures 5 and 6). However, prior to claiming specificity it is necessary to test the expression of each miRNA on an extended number of MS patients and controls by at least two independent methods.

Currently, only one additional study investigated miRNA expression in patients with MS [3]. Otaegui and coworkers analyzed four patients with MS during a relapse and nine MS patients during remission. Patients during relapse and patients during remission were compared with each other and with eight healthy individuals using a non-parametric ranking method called Symmetrical Uncertainty (SU). In total Otaegui et al. analyzed the expression of 364 miRNAs in peripheral mononuclear cells (PBMC). In our study, we analyzed all 866 miRNAs and miRNA star sequences deposited in the miRBase version 12.0 and ranked the miRNAs according to their AUC value for the classification MS versus healthy. Comparing the results of Otaegiu et al. with our results, we found an overlap of seven miRNAs which had high information content in both studies. Of these seven miRNAs, the four miRNAs hsa-miR-509-3-5p, hsa-miR-214, hsa-miR-34c-3p, and hsa-miR-509-5p show an AUC value lower than 0.3 in our study, indicating a higher expression in healthy individuals compared to MS patients. The remaining three miRNAs, namely hsa-miR-328, hsa-miR-30a, and hsa-miR-30e, showed higher expression in the blood cells of MS patients compared to the blood cells of healthy individuals, as indicated by AUC values higher than 0.7.

The small number of miRNAs that are highly informative in both studies may be due to the different experimental approaches used in the two studies. While Otaegui et al. performed quantitative Real Time-PCR we hybridized microarrays with labeled RNA without an initial reverse transcription or amplification step. In addition, our study analyzed more than twice as many miRNAs than the study of Otaegui and colleagues.

Besides the potential role of miRNAs as biomarkers for RRMS, the finding of deregulated miRNA expression in patients with MS suggests that the disease process of MS may result in altered miRNA expression profiles or that deregulation of miRNAs may contribute to the disease process of MS. Among the ten most deregulated miRNAs, hsa-miR-145, hsa-miR-186, and hsa-miR-20b have been found to be deregulated in different types of cancer such as prostate cancer, pancreatic cancer or gastric cancer [13]–[15]. Notably, two of the ten most significantly deregulated miRNAs in our study, namely hsa-miR-142-3p and hsa-miR-223, have previously been found to be differentially expressed in hematopoietic cell lineages. hsa-miR-142-3p is also highly expressed in T-cells [16], [17]. This may be compatible with a role of these miRNAs in haematopoiesis and probably in the immune system. Five out of the ten most deregulated miRNAs (miRNAs hsa-miR-664, hsa-miR-422a, hsa-miR-584, hsa-miR-1275, and hsa-miR-491-5p) have not yet been associated with any specific functions or human diseases.

In conclusion, the data presented in this study demonstrate that miRNA expression profiles differentiate patients with MS from healthy subjects with high accuracy, indicating that miRNAs have the potential to serve as a diagnostic biomarker for RRMS. Future studies should clarify whether these small molecules are also suited for differentiation of specific courses or different pathogenetic subtypes of MS.

## Materials and Methods

### Patients with MS and healthy controls

We included 20 patients with a diagnosis of RRMS according to McDonald criteria [18] as well as 19 healthy controls in our study (see supplemental Table S1). Patients were either treated with glatiramer acetate (n = 9), or interferon-β (n = 10), or did not take any disease-modifying therapy (n = 1). None of the patients had received glucocorticosteroids for at least 3 months before inclusion in the study. All participants of the study have given their written informed consent. Blood from MS patients was obtained during clinical diagnosis. Blood analysis from healthy donors was also performed with written informed consent.

### miRNA microarray screening

About 5 ml blood was collected from study subjects in PAXgene Blood RNA tubes (BD, Franklin Lakes, New Jersey USA). Total RNA was extracted from blood cells using the miRNeasy Mini Kit (Qiagen GmbH, Hilden, Germany) and stored at −70°C. Samples were analyzed with a Geniom Realtime Analyzer (GRTA, febit GmbH, Heidelberg, Germany) using the Geniom Biochip miRNA homo sapiens. Each array contains 7 replicates of 866 miRNAs and miRNA star sequences as annotated in the Sanger miRBase 12.0 [11]. Sample labelling with biotine was carried out by microfluidic-based enzymatic on-chip labelling of miRNAs (MPEA) as described before [19].

Following hybridization for 16 hours at 42°C the biochip was washed automatically and a program for signal enhancement was processed with the GRTA. The resulting detection pictures were evaluated using the Geniom Wizard Software. For each array, the median signal intensity was extracted from the raw data file such that for each miRNA seven intensity values were calculated corresponding to each replicate copy of miRBase on the array. Following background correction, the seven replicate intensity values of each miRNA were summarized by their median value. To normalize the data across different arrays, quantile normalization was applied and all further analyses were carried out using the normalized and background subtracted intensity values.

The raw data and normalized data are available under Gene Expression Omnibus, one of the largest MIAME compatible expression profile resources on the web, under accession GSE17846.

### Statistical analysis

After having verified the approximate normal distribution of the measured data using Shapiro Wilk test, we carried out parametric t-test (unpaired, two-tailed) for each miRNA separately, to detect miRNAs with different expression levels between study groups. The resulting p-values were adjusted for multiple testing by Benjamini-Hochberg adjustment.

To analyze the relationship of the detected miRNAs to miRNAs previously detected in other human diseases we used the “Human miRNA-associated Disease Database (HMDD, [12], http://202.38.126.151/hmdd/login/?next=/hmdd/mirna/md/). In more detail, we built a bipartite graph where nodes correspond either to a miRNA or to a disease. Only edges between miRNA and disease nodes are allowed, where an edge between miRNA *A* and disease *B* means that the miRNA *A* is differentially regulated in disease *B*. Since for MS no deregulated miRNAs have been described so far we added the node “Multiple Sclerosis” to this network and linked it to all miRNAs that were significant deregulated in our analysis.

In addition to the single biomarker analysis and network analysis, classification of samples using miRNA patterns was carried out using Support Vector Machines (SVM) as implemented in the R e1071 package. In particular, different kernel (linear, polynomial, sigmoid, radial basis function) SVMs were evaluated, where the cost parameter was sampled from 0.01 to 10 in decimal powers. The measured miRNA profiles were classified using 100 repetitions of standard 10-fold cross-validation.

To detect the most suitable set of miRNAs that achieves the best discriminatory performance, a feature extraction (subset selection) method relying on t-test p-values has been used. In detail, we decided to use the following stepwise forward filter approach: The *s* miRNAs with lowest p-values in the t-test were computed on the training set in each fold of the cross validation, where *s* was sampled from 1 to 866. The respective subset was used to train the SVM and to carry out the prediction of the test samples. As result, the mean accuracy, specificity, and sensitivity were calculated together with the 95% confidence intervals (95% CI) for each subset size. To check for overtraining we applied permutation tests. Here, we sampled the class labels randomly and carried out classifications using the permuted class labels. All statistical analyzes were performed using R.

## Supporting Information

Figure S1 (0.93 MB PDF)

Figure S2 (0.57 MB PDF)

Table S1 (0.21 MB XLS)
