# Supplementary figures and images for: Multiple Sclerosis: MicroRNA Expression Profiles Accurately Differentiate Patients with Relapsing-Remitting Disease from Healthy Controls
Source: PLoS One. 2009 Oct 13;4(10):e7440. doi: 10.1371/journal.pone.0007440 (PMC2757919; doi:10.1371/journal.pone.0007440)

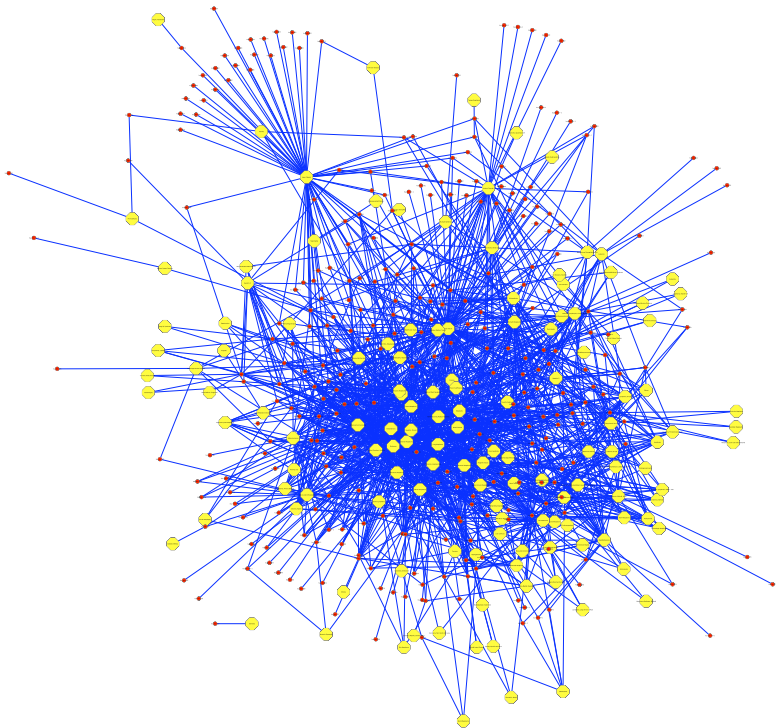

Supplement: Figure S1 — (0.93 MB PDF) [file pone.0007440.s001.pdf]

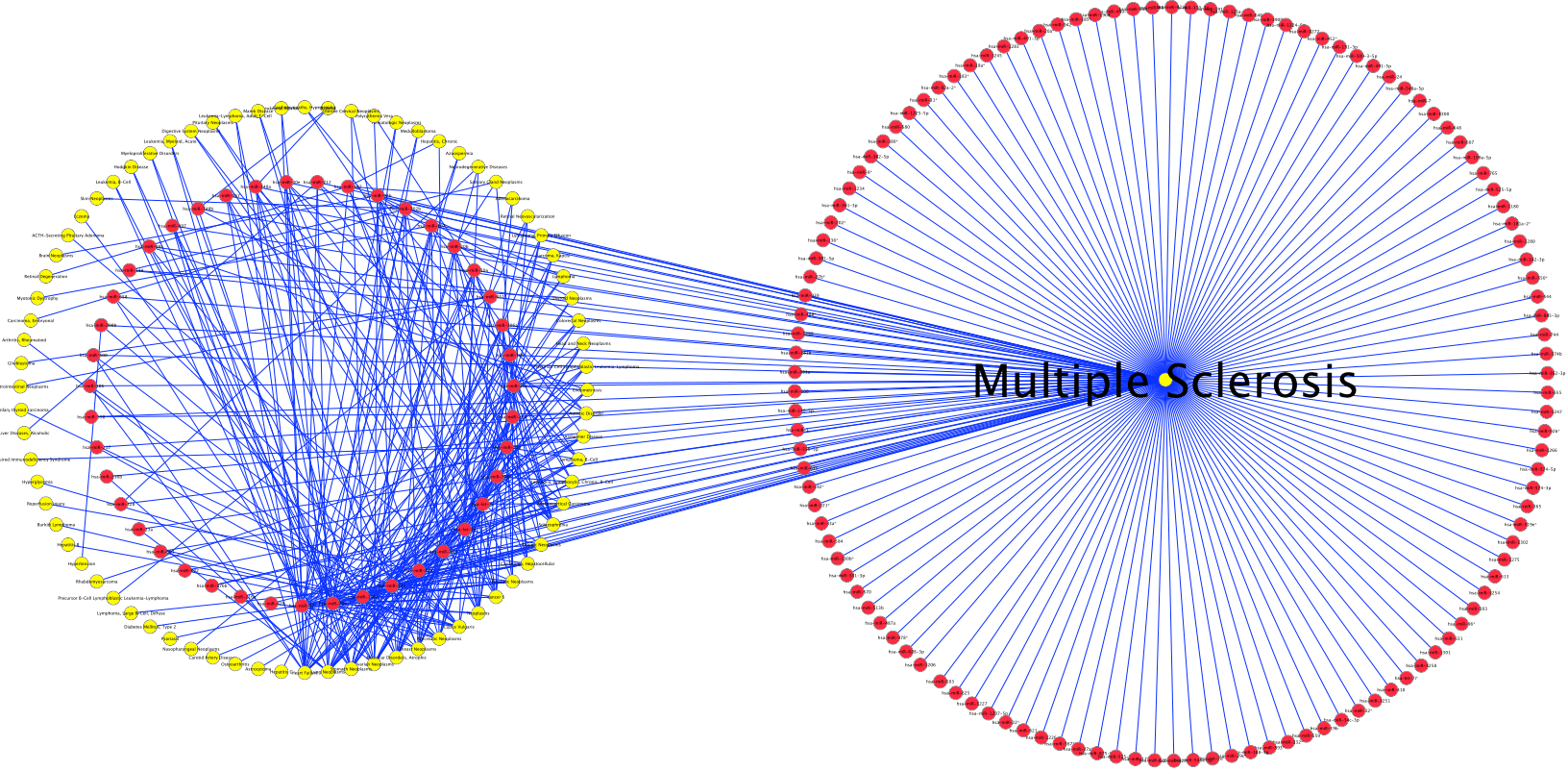

Supplement: Figure S2 — (0.57 MB PDF) [file pone.0007440.s002.pdf]
